# DNA cross-triggered cascading self-amplification artificial biochemical circuit†
†Electronic supplementary information (ESI) available. See DOI: 10.1039/c4sc03225j



**DOI:** 10.1039/c4sc03225j

**Published:** 2014-11-07

**Authors:** Ji Nie, Ming-Zhe Zhao, Wen Jun Xie, Liang-Yuan Cai, Ying-Lin Zhou, Xin-Xiang Zhang

**Affiliations:** a Beijing National Laboratory for Molecular Sciences (BNLMS) , Key Laboratory of Bioorganic Chemistry and Molecular Engineering of Ministry of Education , College of Chemistry , Peking University , Beijing , P. R. China . Email: zhouyl@pku.edu.cn ; Email: zxx@pku.edu.cn ; Fax: +86 10 6275 4680 ; Tel: +86 10 6275 4112

## Abstract

The construction of compact and robust artificial biochemical circuits based on nucleic acids can help researchers to understand the essential mechanisms of complex biological systems, and design sophisticated strategies for various requirements. In this study, a novel DNA cross-triggered cascading self-amplification artificial biochemical circuit was developed. Once triggered by trace amounts (as low as 2 amol) of either of two fully independent oligonucleotide factors under homogeneous isothermal conditions, the circuit simultaneously amplified both factors by 10^5^–10^7^ fold, which was proved using mass spectrometry. The compact and robust circuit was successfully used to construct a multi-input Boolean logic operation and a sensitive DNA biosensor based on the dual-amplification of both the target and reporter. The circuit showed great potential for signal gain in complicated molecular programming, and flexible control of nucleic acid nanomachines in biochemical network systems and nanotechnology.

## Abstract 2

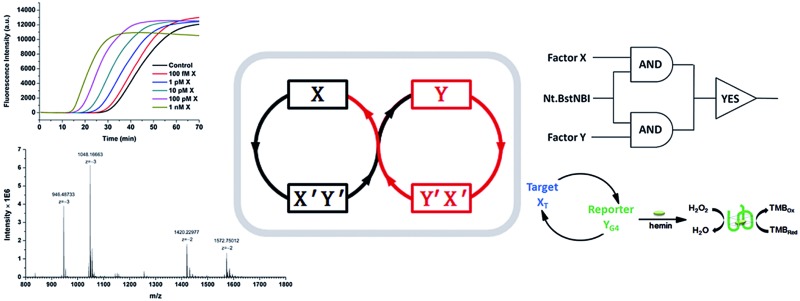
A novel DNA cross-triggered cascading self-amplification artificial biochemical circuit can be triggered by either of two fully independent oligonucleotide factors (∼2 amol) and amplify both of them by 10^5^–10^7^ fold.

## Introduction

Just as electric circuits are required for the engineering of electro-mechanical devices, biochemical circuits can perform complicated biochemical reactions and regulate pathways *via* invisible wire lines. The development of artificial biochemical circuits can help researchers to understand the essential mechanisms of complex biological systems and design sophisticated strategies for the accurate regulation of nanodevices, biochemical reactions or gene expression.^1,2^ They enable the unprecedented control of molecular reactions in the bioengineering and biochemical industries.

Nucleic acid (NA) is a versatile material for the flexible, rational and predictable construction of artificial biochemical circuits with desired dynamic behaviors and rules.^3,4^ NA-based circuits have been reported for logic gate operation,^5^ scaling up DNA computation,^6^ and molecular programming.^7^ Generally, non-enzymatic toe-hold mediated strand displacement reactions are involved in cascading and signal gain.^8^ Tan *et al.* ^9^ introduced an anti-protein aptamer into a circuit to describe an aptamer–thrombin-based logic circuit with a toe-hold mediated threshold controller and an inhibitor generator for the manipulation of protein activity. Besides non-enzymatic approaches, there are many enzyme-based biochemical circuits, such as transcriptional oscillators^10^ and bistable switches.^11^ Fijii and Rondelez^12^ reported a synthetic DNA system that involved programmable interaction under the control of three enzymes to reproduce the predator–prey molecular ecosystem.

Different NA-based artificial biochemical circuits are based on various NA molecular components with specific structures and interaction mechanisms. In the toe-hold mediated strand displacement process, since the relative stability of duplex DNA forms the foundation of dynamic transformation, the sequence design with multi-stranded NA complexes and complicated tuning are huge challenges, especially for some large circuits.^6^ To overcome these challenges, compact, robust, and multi-factor regulated circuits, which enable a more sophisticated representation and exploration of molecular processes, are in great demand. In addition to this, independent circuit-modules are more likely to be combined and stacked in larger networks with comprehensive functions. Cross-catalyzed self-replication circuits can establish an intimate and methodical relationship between two factors such as DNAs,^13^ deoxyribozymogens,^14^ ribozymes^15^ and peptide nucleic acids (PNA),^16^ in an autonomous and compact manner. Most previous studies used chemical template ligation,^13^ ribozyme-mediated anabolic ligation replication,^15^ destabilizing abasic lesion assisted ligase chain reaction,^17^ and DNAzyme-mediated catabolic cross-cleavage^14^ to investigate the probable behaviors and properties of these genetic elements in the prebiotic sequence evolution.^18^ Joyce *et al.* ^19^ developed a theophylline and flavin dependent crossed aptazyme dual sensor system, which established a bridge between exploring the origins of life and developing useful biochemical analysis technologies. However, very few of these crossed self-amplification circuits are adequately effective and flexible to realize magnification towards completely different oligonucleotides instead of two complementary strands. Moreover, the kinds of available ribozyme, deoxyribozyme and aptazyme affiliated pairs are limited and they do not significantly contribute to direct, feasible, reliable and sensitive bioanalysis and application.

Herein, using only two DNA templates and two enzymes to perform isothermal autonomous cascading, a novel DNA cross-triggered self-amplification artificial biochemical circuit was developed. In this circuit, two independent oligonucleotide factors are involved and either of the two can sensitively trigger the rapid and tremendous cascading amplification of both of them. The circuit can act as a smart DNA module for the construction of multi-input related logic operations or dual-amplification NA biosensors.

## Results and discussion

**Scheme 1:**
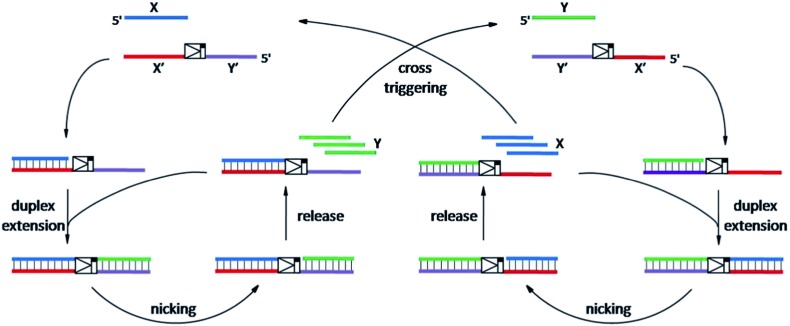
The principle of the cross-triggered cascading self-amplification artificial biochemical circuit.

**Fig. 1:**
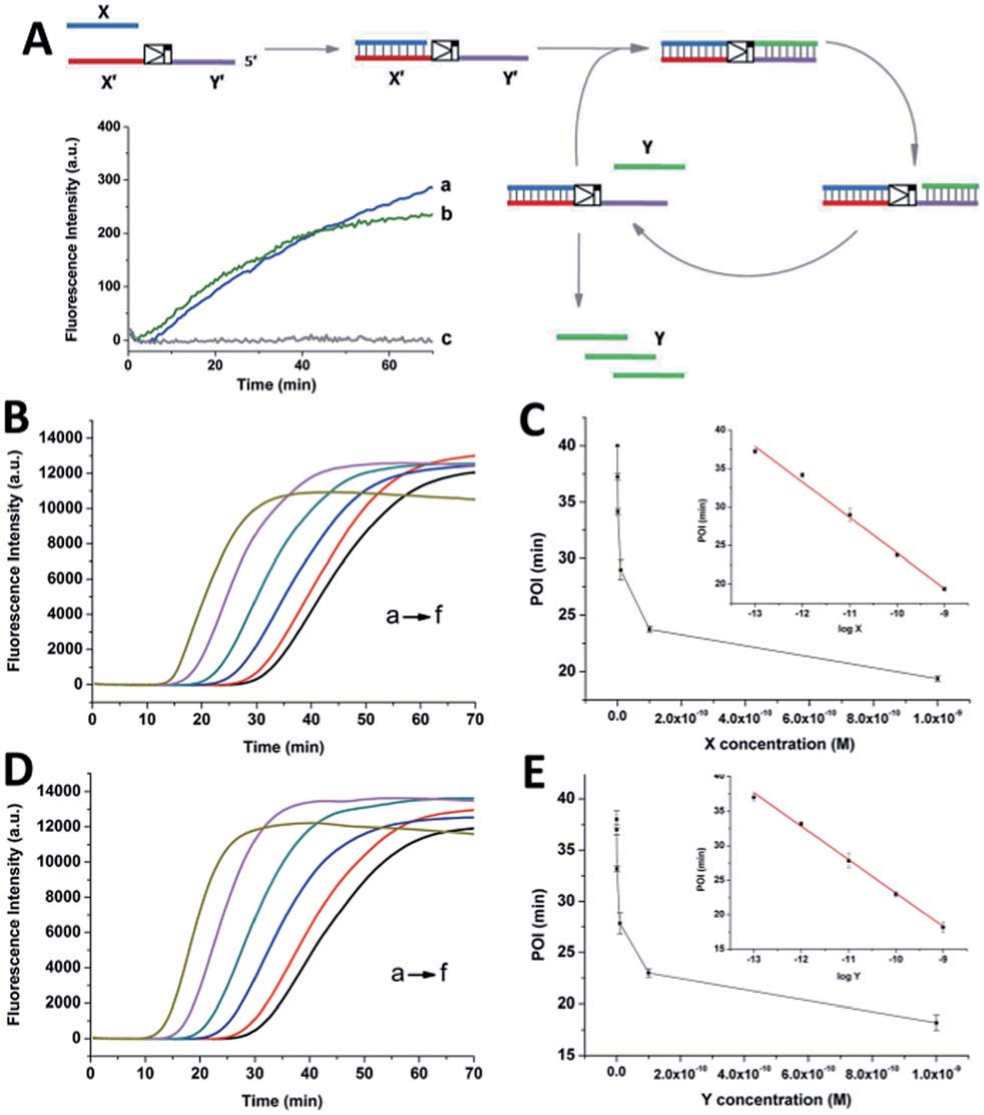
(A) The strand displacement amplification (SDA) circuit triggered by the oligonucleotide factor X. The factor Y is created in a simple linear amplification manner. (The same circuit with factor Y priming Y′X′ is not displayed.) The insert illustrates the fluorescence monitoring of the X/X′Y′ linear circuit triggered by 1 nM X (a) and the Y/Y′X′ circuit triggered by 1 nM Y (b). Line c represents the control experiment performed in the absence of X or Y. (B) and (D) are the real-time fluorescence curves of the cross-triggered cascading self-amplification circuit triggered by different concentrations of the factor X and Y, respectively ((a) 1 nM, (b) 100 pM, (c) 10 pM, (d) 1 pM, (e) 100 fM, (f) 0). (C) and (E) display the relationships between the POI values and the concentrations of factor X and Y, respectively. Experimental conditions: 100 nM X′Y′, 100 nM Y′X′, 0.4 U μL^–1^ Nt.BstNBI, and 0.05 U μL^–1^ Vent (exo-) polymerase. In the strand displacement experiment (A), the unrelated template is removed from the corresponding reaction system. Error bars: standard deviation (SD), *n* = 3.

As shown in Scheme 1, oligonucleotides X (10 nt) and Y (9 nt) represent the two independent factors. Two templates (*i.e.*, X′Y′ and Y′X′) were designed with complementary sequences of X and Y, separated by the complementary sequence for the nicking recognition and cleavage region (5′-GAGTCNNNN↓N-3′, the arrow indicates the cleavage site). Once the trigger factor X primes the template X′Y′, the primer/template X/X′Y′ is polymerized by Vent (exo-) polymerase. Then, Nt.BstNBI can cleave a phosphodiester bond to release the new oligonucleotide factor Y from the XY/X′Y′ double-stranded structure *via* melting-off or strand displacement. The remaining duplex can continue growing and releasing Y constantly in a linear amplification manner. The created factor Y acts as the trigger of the other half of the artificial circuit and hybridizes with the template Y′X′ to form Y/Y′X′. Following polymerization and nicking, the factor X can be continuously produced, which in turn hybridizes with new X′Y′ and initiates a new cycle *via* the same protocol. It is obvious that using factor Y instead of X as the trigger can initiate the reactions of the circuit and result in a cascade of oligonucleotide factor production. Actually, this cross-triggered cascading circuit can be disassembled into two simple isothermal strand displacement amplification (SDA)^20^ linear circuits, *i.e.*, X/X′Y′ and Y/Y′X′ (Fig. 1A). The oligonucleotide product of one SDA linear circuit is the trigger for the counterpart SDA linear circuit and *vice versa*. It was anticipated that the cross-triggered cascading process could drive the exponential accumulation of both independent factors. When all the templates are occupied by their corresponding factors, the exponential manner will convert into linear manner.

SYBR-Green I, an intercalating fluorescent dye, was employed to monitor the generation of partial or complete double-stranded DNA (dsDNA) in the circuit using a real-time fluorescence polymerase chain reaction (PCR) machine. Since the circuit could be divided into two SDA reactions, the signal increase of the cascading circuit was compared with that of a simple SDA circuit. In an individual SDA system, the template was initially occupied by the X or Y factor and converted from single-stranded DNA (ssDNA) to partial dsDNA. Then, it was polymerized into complete dsDNA and the oligonucleotide factor Y or X was released. The fluorescence intensity is proportional to the amount of dsDNA regions. It was found that 1 nM X or Y only triggered a slight increase in the fluorescence signal in the linear circuit over 70 min (insert fluorescence curves in Fig. 1A). By contrast, under the optimized conditions (Fig. S1–S3†), when the crossed cascading system was triggered using different concentrations of factors X (Fig. 1B and C) or Y (Fig. 1D and E), the fluorescence signal increased in a sigmoidal manner with a sharp rise observed irrespective of which triggering factor was used. All the signals finally reached a plateau when all ssDNA templates were converted into dsDNA. Not only was the shape of the real-time curve different from that obtained in the SDA circuit, but the signal intensity was remarkably stronger, implying that the two individual SDA circuits were successfully linked by the enormous cross-generation of factors X and Y. In agreement with the alternate and well-ordered rule, automatic circular cascading was achieved. The point of infection (POI),^21^ which is defined as the time point corresponding to the maximum gradient of the slope of the sigmoidal fluorescence curves, was adopted to describe the triggering performance of the circuits. A good linear relationship was observed between the POI values and the logarithm of the concentration of the trigger which ranged from 100 fM to 1 nM (2 amol to 20 fmol). The fitted equation for trigger X is POI = –22.2 – 4.62 log *X* (*R* ^2^ = 0.993) and for trigger Y it is POI = –25.3 – 4.84 log *Y* (*R* ^2^ = 0.995). The results indicated that factor X or Y, at concentrations as low as 2 amol, could trigger the cascading circuit accurately and result in significant signal responses.

**Fig. 2:**
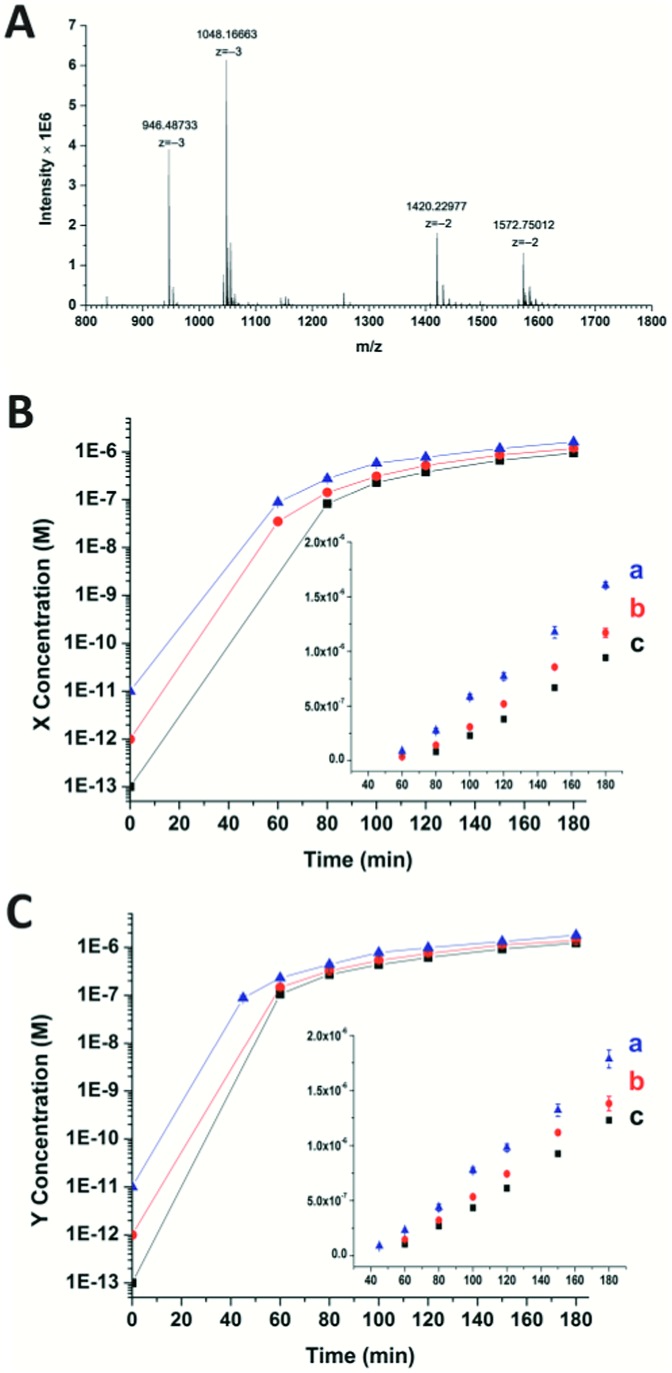
(A) Mass spectrum of the oligonucleotides X and Y produced in the circuit. (B) and (C) display the amounts of X and Y produced in the circuit when triggered by different concentrations of X ((a) 10 pM, (b) 1 pM, (c) 100 fM). The initial concentration is not able to be measured by the mass spectrometer. For the triggering factor, the added amount is used as the initial concentration. For the generated factor Y in (C), it is assumed that the added trigger X can prime the relevant template and generate an equal amount of factor Y in the first cycle in split-second time. Error bars: SD, *n* = 3.

To confirm the generation of the two oligonucleotides and the self-amplification efficiency of our artificial circuit, liquid chromatography electro-spray ionization tandem mass spectrometry (LC-ESI-MS) was performed. From the MS spectrum, factor X [*m*/*z* 1048.16630 (–3) and 1572.75012 (–2)] and factor Y [*m*/*z* 946.48733 (–3) and 1420.22977 (–2)] produced in the artificial circuit could be easily identified (Fig. 2A). X and Y after significant amplification (>50 nM, the limits of detection of the oligonucleotides X and Y obtained by LC-MS are shown in Fig. S4†) at different incubation times could be detected. As shown in Fig. 2B and C, when the circuit was triggered by different concentrations of X, remarkable generation of both X and Y occurred. The amplification by several orders of magnitude was obtained within the first 40 min to 1 h. Then, the amplification trends illustrated in the curves switched to a less effective amplification phase (*i.e.*, linear amplification) since all the templates were used. The same phenomenon was observed when the circuit was triggered by different amounts of Y (Fig. S5†). Namely, regardless of which of the triggers, X or Y, was used, the result was rapid generation of both factors up to micromolar levels with high efficiency (∼10^5^–10^7^ fold). Therefore, the result of the MS was consistent with the principle of the cross-triggered self-amplification mentioned above. Different from some previous isothermal NA amplification strategies, in which two complementary oligonucleotides (exponential SDA^20^ and helicase-dependent amplification^22^) or a single oligonucleotide (isothermal exponential amplification reaction^23^) were amplified, our circuit achieved the significant cascading generation of two fully independent oligonucleotides simultaneously. Considering the amplification folds and reaction time, this circuit is more highly effective than most other cross-triggered self-amplification circuits.^14,15,19^

**Fig. 3:**
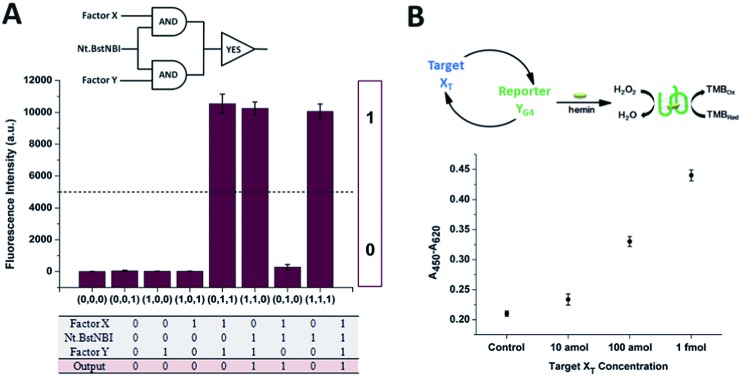
(A) The equivalent electronic circuit, the fluorescence intensity for different combinations of the three inputs and a truth table for the logic operation. Experimental conditions: 375 μM dNTPs, 100 nM X′Y′, 100 nM Y′X′, and 0.05 U μL^–1^ Vent (exo-) polymerase are treated as the work units. The three inputs are 0.4 U μL^–1^ Nt.BstNBI, 1 nM X, and 1 nM Y. The output signal is obtained at 30 min and is monitored by real-time fluorescence PCR. The threshold is set at 5000 a.u. (B) A schematic illustration and quantification of the performance of the dual-amplification (target X_T_ and reporter Y_G4_) biosensor *via* the cross-triggered cascading circuit. Error bars: SD, *n* = 3.

The construction of large DNA-based circuits would satisfy the need for sophisticated functions and applications in biochemical networks and molecular programming. The compact cross-triggered cascading circuit with remarkable amplification efficiency is suited for signal gain in modular molecular design. Taking advantage of the two independent factors acting as a stimulus, the strategy could be used in the switching on/off of molecular devices or nanomachines. Moreover, it could help us to perform multi-input Boolean logic operations that carry and transfer information and produce logical outputs. Two templates, a polymerase and substrate dNTPs, were used as the work unit in the AND gate, while factor X or Y and Nt.BstNBI acted as the two inputs (Fig. 3A). The outputs of the two AND gates (*i.e.*, the generation of a large amount of partial and complete dsDNA) were translated by a YES gate, in which the fluorescent dye SYBR-Green I was used as the work unit. As a result, a high fluorescence readout was obtained as the logic output. Thus, an integrated three-input logic operation was achieved and the results quite agreed with our design.

Additionally, the control of NA-based circuits can be connected with molecular sensors and actuators. Towards these goals, the artificial circuit could be directly applied through treating the target DNA as the trigger factor, or through other transduction events (*e.g.*, target/aptamer affinity interaction) that create the trigger DNA. Since the counterpart factor in the circuit is designed as the reporter molecule, both the signal trigger and signal readout obey the cross cascading mechanism and can be simultaneously and enormously self-amplified, which opens up a new strategy for the dual-amplification of both target and reporter (or intermediate and reporter) in biosensors. As a proof of concept, a smart dual-amplification DNA sensor was tested for the target DNA X_T_ *via* the G-quadruplex sequence Y_G4_ as the reporter (Fig. 3B). G-quadruplex can form a G-quadruplex/hemin DNAzyme complex, which performs peroxidase-mimicking activity and has been widely used as a label-free tag for signal readout in NA-based biosensors.^24^ The recognition of the target X_T_ was converted into the generation of the reporter Y_G4_, which gave quantitative colorimetric signal readouts *via* TMB (4,4′-diamino-3,3′,5,5′-tetramethylbiphenyl)-H_2_O_2_ coloration. Through the isothermal cascading self-amplification of both the target and reporter in the cross circuit, an amount of target X_T_ as low as 10 amol could be sensitively detected, demonstrating a performance better than many homogeneous DNA sensors (Table S2†). When random DNA oligomer sequences were employed in the reaction at 100 times higher concentration, no difference was observed compared to the negative control (Fig. S6†), which confirmed the specificity toward the target X_T_. Some of the previously reported dual-amplification sensing modes were focused on target recycling^25,26^ and magnification of signal readout elements *via* hybridization chain reaction,^27^ rolling circle amplification^28^ and nanoparticles.^29^ In our system, target and reporter oligonucleotides are directly generated with perfect symmetrical equality. The in-one-tube dual-amplification homogeneous strategy could be adopted to meet the requirements of accessible, simple and sensitive NA-based point-of-care assays in clinical diagnosis.

## Conclusion

Taking advantage of the rapid extension and nicking, carried out by the polymerase and nicking enzyme, respectively, and of the two tunable templates directed towards changeable oligonucleotide factors, a novel DNA cross-triggered self-amplification artificial biochemical circuit could be easily, reliably and flexibly developed. Regardless of which of the two oligonucleotide factors triggered the circuit, a 10^5^–10^7^ fold amplification of both independent factors was achieved at the same time. The artificial biochemical circuit was successfully adopted for the construction of a three-input Boolean logic operation for information carrying and transferring, and a smart G-quadruplex based target/reporter dual-amplification colorimetric biosensor. Furthermore, the compact cascading circuit has great potential for application in modular molecular design to establish biochemical networks or nanodevices with sophisticated functions in biochemical analysis and engineering.
