# Supplementary material for: DNA cross-triggered cascading self-amplification artificial biochemical circuit
Source: Chem Sci. 2014 Nov 7;6(2):1225–9. doi: 10.1039/c4sc03225j (PMC5811080; doi:10.1039/c4sc03225j)
Supplement: Supplementary file 1 [file SC-006-C4SC03225J-s001.pdf]

Electronic Supplementary Material (ESI) for Chemical Science.
This journal is © The Royal Society of Chemistry 2014

Supporting Information

DNA Cross-Triggered Cascading self-amplification Artificial Biochemical Circuit

Ji Nie, Ming-Zhe Zhao, Wen Jun Xie, Liang-Yuan Cai, Ying-Lin Zhou and Xin-Xiang Zhang**

Beijing National Laboratory for Molecular Sciences (BNLMS), Key Laboratory of Bioorganic Chemistry and Molecular Engineering of Ministry of Education, College of Chemistry, Peking University, Beijing 100871, China

Experimental Section

Reagents and Materials

All HPLC-purified DNA oligonucleotides (listed in Table S1) were synthesized by Sangon Biological Engineering Technology & Services Co., Ltd (Shanghai, China). The vent (exo-) DNA polymerase and nicking endonuclease Nt.BstNBI were purchased from New England Biolabs (Beverly, MA). Deoxynucleotide mixture (dNTPs) was bought from Takara Biotechnology Co. Ltd (Dalian, China). SYBR-Green I (20×) in Dimethyl Sulphoxide (DMSO) was obtained from Xiamen Zeesan Biotechnology (Xiamen, China). N,N-Dimethylbutylamine and acetic acid (HAc) were bought from Sigma-Aldrich (St.Louis, MO, USA). LC-MS grade acetonitrile (ACN) and water were purchased from J.T.Baker (Center Valley, PA). Hydrogen peroxide (H_2O_2) was bought from Beijing Chemical Reagent Company (Beijing, China). TMB·2HCl (TMB: 4,4'-diamino-3,3',5,5'-tetramethylbiphenyl) was obtained from Ameresco (USA). Hemin was purchased from Sigma-Aldrich (St.Louis, MO, USA). A 5 mM stock solution (in DMSO) was stored at -20℃. All other reagents were at least of analytical reagent grade.

Cross-Triggered Cascading self-amplification Circuit

The circuit was prepared separately as part A and part B solutions. Part A consisted of X′-Y′ and Y′-X′ template, trigger X or Y, Nt.BstNBI buffer and dNTPs. Part B consisted of the nicking endonuclease Nt.BstNBI, vent (exo-) DNA polymerase and ThermoPol buffer. Part A and Part B were mixed immediately containing X′-Y′ template (100 nM), Y′-X′ template (100 nM), different concentrations of X or Y, Nt.BstNBI (0.4 U/μL), vent (exo-) DNA polymerase (0.05 U/μL), dNTPs (375 μM), SYBR-Green I 0.5× , 0.5× Nt.BstNBI buffer (25 mM Tris-HCl, pH 7.9, 50 mM NaCl, 5 mM $MgCl_2$, 50 μg/mL BSA) and 1 ×ThermoPol buffer (20 mM Tris-HCl, pH 8.8, 10 mM KCl, 10 mM $(NH_4)_2SO_4$, 2 mM $MgCl_2$, 0.1% Triton X-100). The 20 μL mixture was incubated and monitored at 55℃ by CFX96^{TM} Real-Time PCR detection system (Bio-Rad, USA). The circuit product preparing for LC-ESI-MS was carried out by TC-512 PCR (TECHNE, UK) without SYBR-Green I. After incubation at 55℃ for different time, the reaction mixtures were heated to 80℃ for 20 min and stored at 4℃. To perform LC-ESI-MS quantification, a 20 μL product solution was mixed with 2 μl 10 μM internal standard IS-ssDNA for normalization.

LC-ESI-MS Quantification of Circuit Products

The LC-ESI-MS quantification of produced X and Y in the circuit was carried out on Agilent 1200 HPLC and 6320 ion trap Mass spectrometer (Palo Alto, CA, USA). Hypersil GOLD 100×2.1 mm, 3 μm particle size, C-18 reverse column (Thermo Fisher, MA, USA) preheated to 30℃ was involved in separation. Injection volume was 3 μL. Flow rate was set as 0.2 mL/min. Elution was initiated with 95% mobile phase A (5 mM dimethylbutylamine acetate, DMBAA aqueous solution) and 5% mobile phase B (5 mM DMBAA in ACN) over 1.5 min for desalting. Phase A was ramped from 5% to 80% over 8.5 min and hold for another 5 min. Then the elution condition was back to the initial one. The electrospray was under -3300 V assisted with 30 psi nebulizer gas, while the dry gas flow rate was 7 L/min and the temperature was set to 325℃. The MS detection range was set from 850 to 1200 m/z. LC-ESI-MS spectrum of oligonucleotides produced in circuit (Figure 2A) was performed on Dionex Ultimate 3000 UHPLC and Thermo Q-Exactive mass spectromer (Thermo Fisher, MA, USA).

Smart Dual-amplification biosensor via Cross-Triggered Circuit

Part A consisted of $X_T'Y_{G4}'$ and $Y_{G4}'X_T'$ template, target X_T, Nt.BstNBI buffer and dNTPs. Part B consisted of Nt.BstNBI, vent (exo-) DNA polymerase and ThermoPol buffer. A 10 μL mixture of Part A and Part B containing different concentrations of X_T, $X_T'Y_{G4}'$ template (200 nM), $Y_{G4}'X_T'$ template (200 nM), Nt.BstNBI (0.4 U/μL), vent (exo-) DNA polymerase (0.05 U/μL), dNTPs (375 μM), 1 ×ThermoPol buffer and 0.5 ×Nt.BstNBI buffer was incubated at 55℃ for 90 min and then stored at 4℃.

The reaction solution was mixed with 4 μL 50 μM hemin and 14 μL 1 ×ThermoPol buffer. A 5 μL resulting solution dropped into 96-well plate. The chromogenic reaction was started by the adding of 100 μL TMB-H_2O_2 solution (0.12 mg/mL TMB·2HCl, 18 mM H_2O_2, 0.1 M NaH_2PO_4-Na_2HPO_4, pH 6.0). Peroxidase-mimicking G-quadruplex/hemin DNAzyme can catalyze H_2O_2-mediated oxidation of TMB, which showed significant color change to blue. After 3 minutes incubation at room temperature (avoiding direct light exposure), the reaction was terminated by 50 μL 2 M H_2SO_4 (the color of the solution changed into yellow). Then absorption signals (A_{450}-A_{620}) were recorded by Thermo Scientific Multiskan FC Microplate Photometer (USA) via dual wavelengths mode.

Table S1 DNA sequences used in this work

Name	Sequence(5'- to 3'-)
X′Y′	CACCTTAGTTCCAGACTCTCCTACGACTG-P
Y'X'	CCTACGACTGTCCAGACTCTCACCTTAGT -P
X	CAGTCGTAGG
Y	ACTAAGGTG
IS-ssDNA	CCCTAA
Target X_T	TGAGGTAGTAGGTTGTATAGTT
Random I	GGAATAACATGACCTGGATGCA
Random II	GACTGTTTATAGCTGTTGGAAG
$X_T'Y_{G4}'$	TCCCTCCCTCCCTCCCAGTCCAGACTCTAACTATACAACCTACTACCTCAA-P
$Y_{G4}'X_T'$	AACTATACAACCTACTACCTCATCCAGACTCTTCCCTCCCTCCCTCCCAGA-P

Table S2 Comparison of different homogeneous DNA sensors

System	Method	Sensitivity	Ref.
Isothermal circular strand displacement polymerization	electrochemistry	100 pM	[1]
Os[(bpy)2(dppz)]$^{2+}$ probe and ExoIII-aided target recycling	electrochemistry	2.5 nM	[2]
Ru(II) complex functionalized grapheme oxide	electrochemiluminescence	100 pM	[3]
Hybridization chain reaction and AuNPs	colorimetry	50 pM	[4]
G-quadruplex based hybridization chain reaction	colorimetry	7.5 nM	[5]
Template-dependent extension based isothermal amplification	fluorescence	10 pM	[6]
Autonomous Zn^{2+}-dependent ligation DNAzyme machinery	fluorescence	10 pM	[7]
Fluorescence Quenching of Carbon Nitride Nanosheet	fluorescence	81 pM	[8]
Cross-triggered cascading self-amplification circuit	colorimetry	1 pM (10 amol in 10 μL)	this work

The optimization of fluorescence monitoring

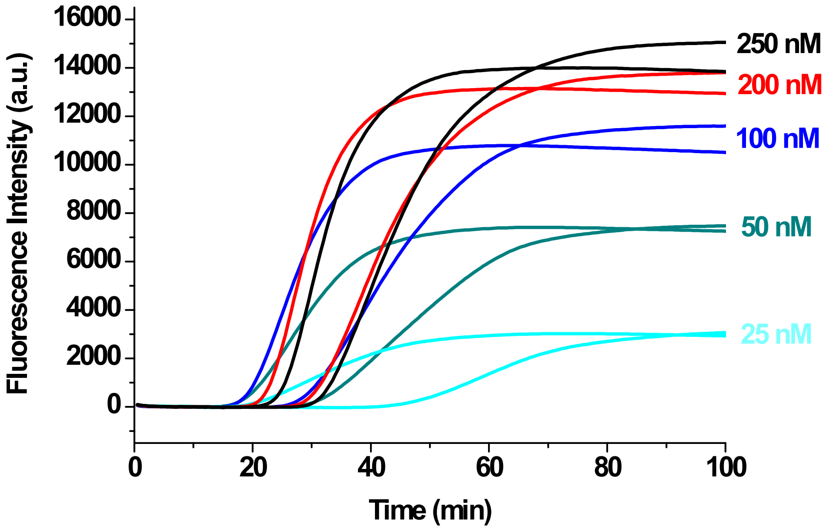

Figure S1 The optimization of template concentrations. The amount of both X′-Y′ and Y′-X′ was: 25 nM, 50 nM, 100 nM, 200 nM and 250 nM. The real-time fluorescence curves of cross-triggered cascading circuit were triggered by 50 pM X (left curves) and the blank (right curves) under the condition described in Experimental Section. 100 nM X′-Y′ and 100 nM Y′-X′ were selected as optimal conditions.

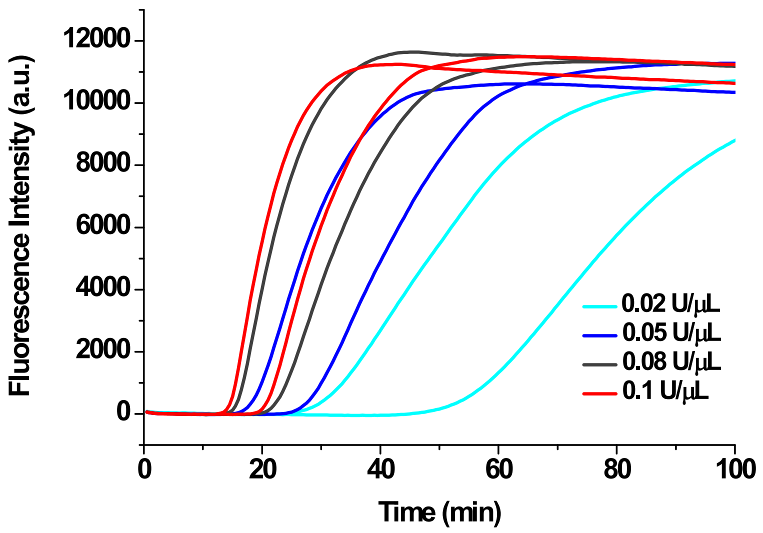

Figure S2 The influence of Vent (exo) polymerase concentration (0.02, 0.05, 0.08, 0.1 U/μL). The real-time fluorescence curves of cross-triggered cascading circuit were triggered by 50 pM X (left curves) and the blank (right curves) under the condition described in Experimental Section. 0.05 U/μL Vent (exo) polymerase was chosen to be optimal condition.

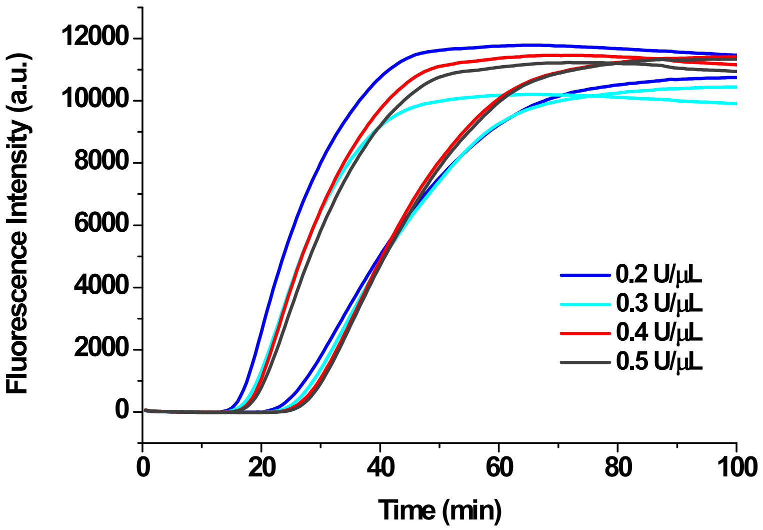

Figure S3 The effect of the amount of Nt.BstNBI (0.02, 0.05, 0.08, 0.1 U/μL). The real-time fluorescence curves of cross-triggered cascading circuit were produced by 50 pM X (left curves) and the blank (right curves) under the condition described in Experimental Section. 0.4 U/μL Nt.BstNBI was adopted.

Calibration Curves of factors X and Y

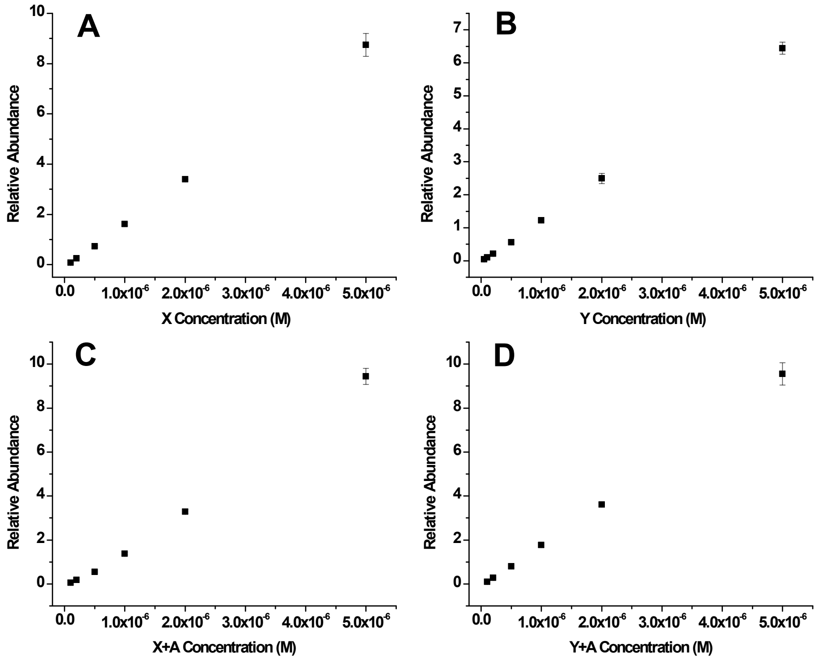

Figure S4 Calibration curves of four kinds of short ssDNA: A. factor X, B. factor Y, C. DNA X+A (adenine), D. DNA Y+A. Relative abundance is the ratio of target signal to the signal intensity of internal standard IS-ssDNA. Error bars: SD, n = 3

The calibration curves were carried out by LC-ESI-MS following the protocol described in experimental section. Different from the factor added, the cleaved oligonucleotide has a phosphate on the 5′-end. The resulting ends of oligonucleotides by vent (exo-) polymerase are the mixture of blunt and single-base 3′ overhangs.[9] (An extra adenine is produced mainly.) We can observe all the four kinds of productions in the reaction solution by MS. Thus, to make the quantification more accurate, the calibration curves for X, Y, X+A and Y+A modified with 5′-P are obtained (Figure S4). The linearity and the limit of detection (LOD) are listed below:

Oligonucleotide	Linear Equation	R^2	LOD
X	$y = 1.734\times10^6x$	0.9992	100 nM
Y	$y = 1.279\times10^6x$	0.9995	50 nM
X+A	$y = 1.831\times10^6x$	0.9929	100 nM
Y+A	$y = 1.889\times10^6x$	0.9990	100 nM

Another thing we should notice is that the products with overhang "A" are not likely to result in any negative effect to the performance of circuit itself. The template sequences we designed have an extra thymine "T" (blod red) between the complementary of triggering recognition (X′, blue region; Y′, green region) and nicking region of Nt.BstNBI (underline). The extra T can form perfect hybridization with 3'-overhang A products, which will not cause any loss of efficiency in cross-triggered cascading circuit. Apart from this, the added "T" in the templates doesn't affect the carrying on of the cascading growth system priming by large amount of created factor X or Y.

X′-Y′: 5′-CACCTTAGTTCCAGACTC**T**CCTACGACTG-P

Y′-X′: 5′-CCTACGACTGTCCAGACTC**T**CACCTTAGT–P

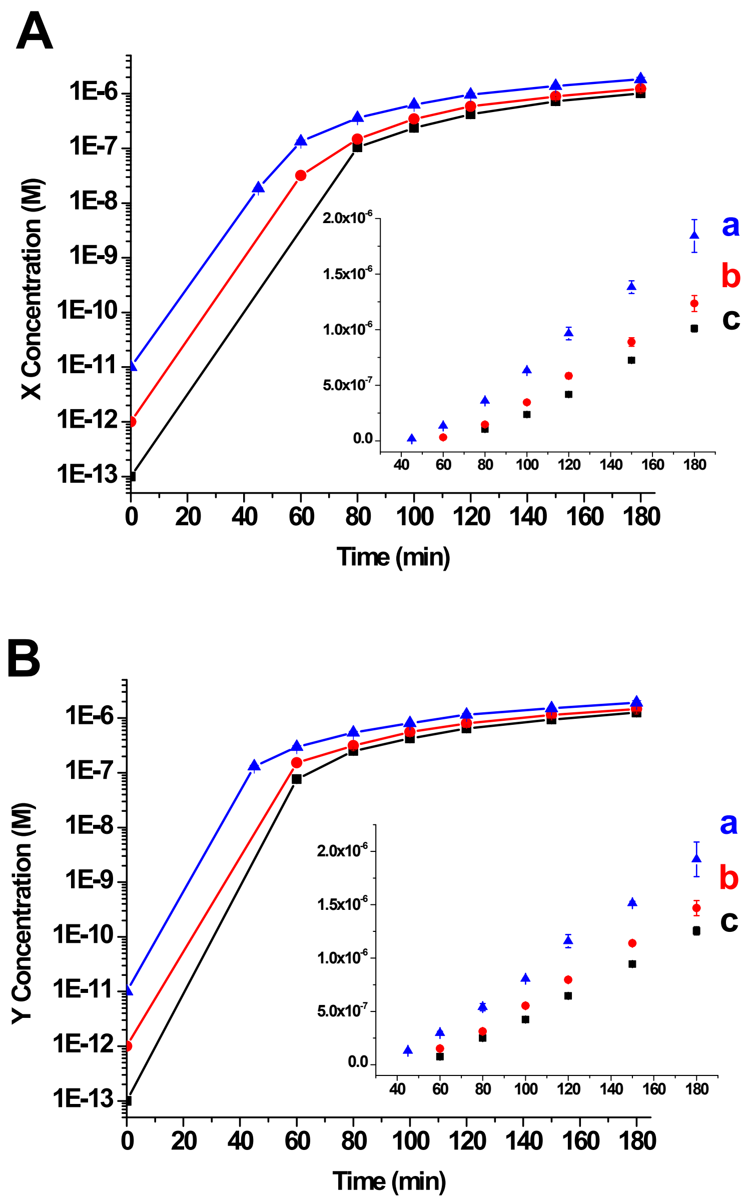

Figure S5 (A) and (B) display the amounts of X and Y produced in the circuit when triggered by different concentrations of Y (a. 10 pM, b. 1 pM, c. 100 fM). The initial concentration is not measurable in the mass spectrometer. For the triggered factor, the added amount is used as the initial concentration. For the generated factor X in (A), it is assumed that the added trigger Y can prime the relevant template and generate the equal amount of the factor X at the first cycle in split-second time. Error bars: SD, n = 3.

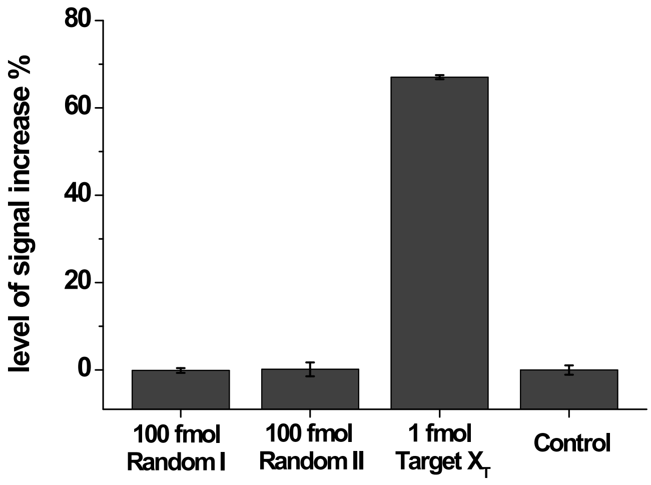

Figure S6 Specificity analysis of smart dual-amplification DNA sensing. Level of signal increase = (Y-N)/N×100%, where Y is the signal triggered by different concentrations of Random I, Random II or target X_T, and N is signal of corresponding negative control (without target X_t).

Reference

[1] F. Xuan, X. T. Luo and I. M. Hsing, *Biosens Bioelectron*, 2012, **35**, 230-234.

[2] R. Miranda-Castro, D. Marchal, B. Limoges and F. Mavre, *Chem. Commun.*, 2012, **48**, 8772-8774.

[3] F. Li, Y. Yu, Q. Li, M. Zhou and H. Cui, *Anal. Chem.*, 2014, **86**, 1608-1613.

[4] P. Liu, X. Yang, S. Sun, Q. Wang, K. Wang, J. Huang, J. Liu and L. He, *Anal. Chem.*, 2013, **85**, 7689-7695.

[5] J. Dong, X. Cui, Y. Deng and Z. Tang, *Biosens Bioelectron*, 2012, **38**, 258-263.

[6] H. Zhou, S.-J. Xie, S.-B. Zhang, G.-L. Shen, R.-Q. Yu and Z.-S. Wu, *Chem. Commun.*, 2013, **49**, 2448-2450.

[7] F. Wang, J. Elbaz and I. Willner, *J Am Chem Soc*, 2012, **134**, 5504-5507.

[8] Q. Wang, W. Wang, J. Lei, N. Xu, F. Gao and H. Ju, *Anal. Chem.*, 2013, **85**, 12182-12188.

[9] *https://www.neb.com/products/m0257-ventr-exo-dna-polymerase*
